# Effects of Imposed Defocus on Inhibitor of DNA-Binding Gene Expression in Chick Posterior Ocular Tissues

**DOI:** 10.3390/cells14231883

**Published:** 2025-11-27

**Authors:** Yan Zhang, Connor Tseng, Abraham Hang, Daniel Sun, Wulian Song, Christine F. Wildsoet

**Affiliations:** 1 Herbert Wertheim School of Optometry and Vision Science, University of California, Berkeley, CA 94720, USA; connor.tseng@berkeley.edu (C.T.); ahang@berkeley.edu (A.H.); dsun8@berkeley.edu (D.S.); wildsoet@berkeley.edu (C.F.W.); 2 Department of Ophthalmology, The 2nd Affiliated Hospital, Harbin Medical University, Harbin 150088, China; songwulian@163.com

**Keywords:** myopia, chick, RPE, gene expression, BMP, Id

## Abstract

Inhibitors of DNA-binding genes (*Id*s) are key downstream targets of bone morphogenetic proteins (BMPs), gene expression of which is differentially regulated in the chick retinal pigment epithelium (RPE) during altered eye growth. The current study examined the effects of optical defocus on the gene expression of *Id1-4* in chick retina, RPE, and choroid after 2 or 48 h of monocular +10 or −10 D lens wear. Defocus-induced differential *Id* gene expression was observed in all three tissues, with defocus sign and treatment duration-related differences. In the choroid, 2 h of +10 D (myopic) defocus induced upregulation of all four of the *Id*s, with this effect also seen with 48 h exposure, for both *Id3* and *Id4* genes. Two hours of +10 D defocus also induced upregulation of both *Id2* and *Id3* in RPE, while 48 h of −10 D (hyperopic) defocus induced downregulation of *Id1*. Gene expression changes in the retina were less predictable. The significant myopic defocus-induced upregulation of expression for all four *Id* genes in the choroid is consistent with previously observed increased *Bmp* gene expression in chick RPE under the same conditions, and offers further supporting evidence for important roles for BMPs and downstream signaling pathways in defocus-driven eye growth regulation.

## 1. Introduction

Uncorrected refractive errors are a leading cause of blindness. Myopia, the most common type of refractive error, has significantly increased in prevalence globally over the past decades, and the continuing rise in the latter is feeding growing concern [1,2], given that myopia has been linked to an increased risk of a number of sight-threatening conditions, including glaucoma and retinal detachment [3,4]. Globally, the percentage of people with myopia is projected to reach approximately 50% by 2050, more than double the 2000 figure of 23% [2]. Refractive errors occur because of mismatches between the length and the focusing power of an eye [5,6], with most myopia being the product of excessive elongation of the posterior vitreous chamber of the eye during development. Thus, when left uncorrected, light rays from distant objects come to focus in front of the retina in the case of myopia, with the opposite being true for hyperopia.

Animal model studies have provided valuable insight into the regulatory pathways and modulatory signals controlling early eye growth [7,8,9,10,11], although there remain many unanswered questions. For example, it is now generally accepted that there is an active emmetropization mechanism, which is responsive to optical defocus and able to regulate eye elongation via visual feedback, to reduce or eliminate existing refractive errors. This mechanism, which is now generally accepted to be regulated locally, i.e., within the eye, can be activated experimentally with the fitting of defocusing lenses to animal subjects. Thus, the use of negative lenses, which shift the plane of focus behind the retina (hyperopic defocus), causes the choroid to thin and the overall rate of axial eye elongation to accelerate, leaving the eye myopic when the lens is removed [12,13]. The opposite is true for positive lenses; the plane of focus is moved in front of the retina (myopic defocus), causing the choroid to thicken and the overall rate of axial eye elongation to slow, leaving the eye hyperopic when the lens is removed. These two experimental paradigms, along with a third form-deprivation paradigm, which also induces myopia, have been used to investigate the underlying local growth-regulating signal pathways, including the effects of such visual manipulations on gene expression patterns in the posterior ocular tissues, i.e., retina, RPE, choroid, and sclera [14,15].

The RPE, which comprises a single layer of cells separating the retina from the choroid, plays a key role in maintaining the health of the outer retina by regulating the local ion balance amongst its many functions [16]. It likely plays a key role as a signal relay for retina-derived growth modulating signals directed at the choroid and sclera, so as to modulate their growth [17]. Our previous studies in chicks support such a role for the RPE and have identified members of the BMP family as potential ocular growth modulators [18,19,20,21,22,23,24]. For example, *Bmp2* gene expression in the RPE was shown to be bidirectionally regulated, with imposed defocus resulting in defocus sign-dependent altered gene expression [24]. Specifically, imposed myopic defocus led to upregulation of RPE *Bmp2* gene expression and hyperopic defocus, to its downregulation. The RPE also recorded a much higher level of *Bmp2* gene expression compared to levels recorded in the adjacent retina and choroid, consistent with it being a component of an as-yet poorly understood paracrine signaling pathway involving BMP2 [24]. RPE *Bmp* gene expression downregulation was also observed in further studies involving diffusers fitted over the eyes of young chicks to induce form-deprivation myopia, adding further support for roles for the RPE and BMPs in ocular growth regulation [20,21].

Inhibitors of DNA binding (*Id1-4*) are recognized to play important roles in the regulation of cell proliferation and cell differentiation [25,26]. As a family, they represent a subgroup of the helix–loop–helix (HLH) transcription factors associated with the transition of a cell cycle from G1 to S phase, achieved through their binding to key regulatory proteins [25,27]. Lacking the adjacent basic region necessary for DNA binding [28,29], the Id-bHLH heterodimers are unable to bind to DNA, thereby inactivating the expression of genes that the bHLH proteins otherwise regulate [26,28,29]. While many different molecular stimuli are known to influence *Id* gene expression, of potential relevance to eye growth regulation are reports of its regulation by BMPs and TGF-βs in various tissues and animal models [30,31,32,33].

The above reports linking BMPs with Ids led to the study reported here, which examined in the chick, changes in the gene expression of key *Ids* in the ocular tissues previously found to show defocus-induced changes in *Bmp* gene expression, i.e., retina, RPE, and choroid [18,19,20,21,22,23,24]. Id1, Id2, Id3, and Id4, which represent the four Ids identified in the chick, were targeted in this study [34,35], which also made use of the same optical defocus paradigms as in our related BMP studies, i.e., positive and negative lenses to impose myopic and hyperopic defocus, respectively [23,24].

## 2. Materials and Methods

### 2.1. Animals and Lens Treatments

White-Leghorn chickens were hatched on-site from fertilized eggs obtained from the University of California, Davis (Davis, CA, USA), and raised under a 12 h light/12 h dark cycle with daytime room illumination averaging 250 lux, as measured with an IL1700 research radiometer (International Light, Inc., Peabody, MA, USA). Chicks were given free access to food and water. At 14 days of age, chicks were fitted with either monocular +10 or −10 D lenses, which were worn for either 2 or 48 h to slow or enhance eye elongation, respectively. The defocusing lenses are custom-designed for use with young chicks; made of polymethyl methacrylate (PMMA) material to minimize their weight and mounted over the eyes of the subjects via velcro support rings for ease of their removal for daily cleaning [36]. The contralateral eyes of all birds served as controls. Age-matched controls that were left untreated were also included in the study (n = 3 to 11 for various treatment conditions). Reference axial length and choroidal thickness biometric data were drawn from an already published study [24]. Experiments were conducted according to the ARVO Statement for the Use of Animals in Ophthalmic and Vision Research, and approved by the Animal Care and Use Committee (ACUC) at the University of California, Berkeley, CA, USA.

### 2.2. Tissue Sample Collection

Retina, RPE, and choroid samples were collected from both eyes of all chicks. The method of tissue collection followed protocols as previously reported by us [23,24]. In brief, chickens were sacrificed, their eyes immediately enucleated, and retina, RPE, and choroidal tissue collected in that order. The retina was collected by gently peeling it from the RPE, with segments of retina contaminated with RPE being discarded. The RPE was then separated from the underlying choroid by gently rinsing the RPE off the choroid with cold 1X phosphate-buffered saline (PBS). Lastly, the choroid was detached from the sclera. All three tissue samples were lysed over ice with RLT buffer (from RNeasy Mini kits, Qiagen, Valencia, CA, USA) and immediately stored at −80 °C for later use.

### 2.3. RNA Purification

RNeasy Mini kits were used to purify total RNA from the retina and RPE samples, while RNeasy Fibrous Tissue Mini Kits (Qiagen) were used to purify total RNA from choroid samples. On-column DNase digestion was then performed on all samples, according to the manufacturer’s protocol. The concentration of RNA in samples and A_260_/A_280_ were measured using a NanoDrop 2000 spectrophotometer (NanoDrop Technologies, Inc., Wilmington, DE, USA).

### 2.4. Real-Time PCR

Eight microliter (uL) RNA samples, diluted to a concentration of 50 ng/uL, first underwent reverse transcription (SuperScript III First-Strand Synthesis System for RT-PCR; Invitrogen, Carlsbad, CA, USA), with gene expression then quantified using QuantiTect SYBR Green PCR Kits (Qiagen) and a StepOnePlus Real-Time PCR System (Applied Biosystems, Carlsbad, CA, USA). The primers were designed using Primer Express 3.0 (Table 1; Applied Biosystems, Foster City, CA, USA). In order to obtain a standard curve and calculate primer efficiency, ten-fold serial dilutions of cDNA were used for each pair of primers, depending on the cDNA concentration. Melt curves were obtained for all genes examined, and each experiment was performed in triplicate. Mean normalized expression (MNE) values were calculated and used to compare expression levels in retina, choroid, and RPE isolated from treated eyes and their fellows (control eyes) [24]. Differential gene expression changes were calculated as treated eye/contralateral control eye × 100%. Mean percentage changes of less than 100% indicate downregulation of gene expression in treated eyes relative to their fellow controls, while greater than 100% indicates relative upregulation of gene expression in treated eyes.

### 2.5. Statistical Analysis

Paired Student’s *t*-tests were used to analyze for each of the four *Id* genes, interocular differences in expression, i.e., between treated and contralateral fellow eyes of lens-wearing chicks and right and left eyes of untreated chicks. The appropriateness of this approach was also confirmed using a Q-Q (Quantile–Quantile) plot combined with the Shapiro–Wilk normality test to review the distribution of the data.

## 3. Results

### 3.1. Id Gene Expression in the Retina and Effects of Lens Treatment

**Figure 1:**
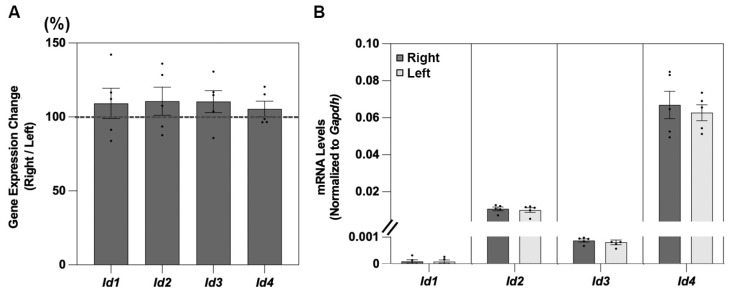
Shown in (**A**) are retinal *Id1-4* gene expression levels, expressed as ratios of results for right versus left eye samples from untreated chicks (%, n = 5), with mRNA levels, normalized to *Gapdh,* for the same right and left eye samples shown in (**B**).

For all four of the genes examined, *Id1*, *Id2*, *Id3*, and *Id4*, expression reached detectable levels in the retinal tissue from untreated chicks (Figure 1 and Table 2). For all four *Id* genes, there was no significant difference in mRNA levels between samples from the right and left eyes of the untreated birds (Figure 1A). However, there were differences between the genes in expression levels, with relatively higher levels recorded for the *Id2* and *Id4* genes compared to the *Id1* and *Id3* genes (Figure 1B).

**Figure 2:**
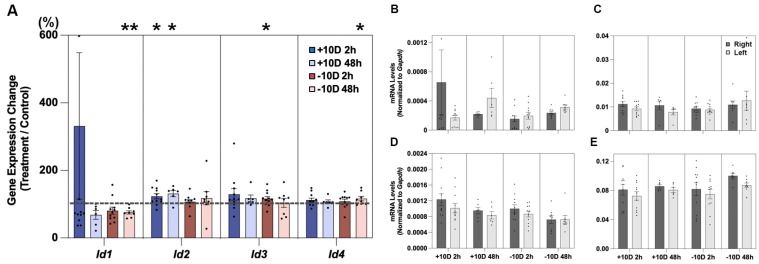
In (**A**), defocus-induced changes in retinal *Id1-4* gene expression are expressed as ratios of results for treated versus fellow eyes (%), after 2 or 48 h of +10 or −10 D lens wear (+10 D, 2 h, n = 11; +10 D, 48 h, n = 6; −10 D, 2 h, n = 11; −10 D, 48 h, n = 8). Retinal mRNA levels, normalized to *Gapdh*, for both treated and control eyes are also shown for *Id1*, *Id2*, *Id3*, and *Id4* ((**B**–**E**), respectively). Note: Differences in Y-axis scales for individual panels are used to accommodate gene-dependent differences in expression levels. * *p* < 0.05; ** *p* < 0.01.

Significant defocus treatment effects were also recorded for all four *Id* genes, although there were gene-related differences (Figure 2A and Table 3). With the +10 D lens, only *Id2* expression was significantly altered, showing upregulation by 123.3 ± 7.8% (*p* = 0.03, n = 11) and 131.2 ± 9.2% (*p* = 0.01, n = 6) after 2 and 48 h of treatment, respectively. In contrast, with the −10 D lens, *Id1*, *Id3*, and *Id4* genes all showed significantly altered expression, with *Id3* showing significant upregulation, by 116.2 ± 6.7% (*p* = 0.04, n = 11), after just 2 h of lens wear. After 48 h of treatment, *Id4* was also upregulated, by 116.3 ± 6.7% (*p* = 0.04, n = 8), while *Id1* was downregulated, to 75.6 ± 4.9% (*p* = 0.007, n = 8). mRNA levels for these same four genes, normalized to *Gapdh*, for retinal samples from treated and fellow eyes are shown in Figure 2B–E and Table 4. Treatment-induced differential *Id* gene expression data, expressed as fold changes (log2), are provided in Supplemental Table S1 and Figure S1.

### 3.2. Id Gene Expression in the RPE and Effects of Lens Treatment

**Figure 3:**
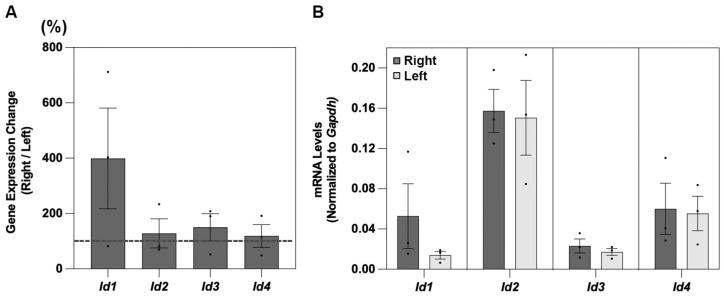
RPE expression levels for *Id1-4* genes, shown in (**A**) as ratios of values for right versus left eyes (%, n = 3), with their mRNA levels, normalized to *Gapdh,* shown in (**B**).

For all four of the genes examined, *Id1*, *Id2*, *Id3*, and *Id4*, expression reached detectable levels in the RPE from untreated chicks (Figure 3 and Table 5). Also, as expected, there were no significant differences in mRNA levels between their right and left eyes for any of the four *Ids*, although there were gene-related differences in expression levels, with *Id2* showing relatively higher expression than *Id1*, *Id3*, and *Id4* (Figure 3B).

**Figure 4:**
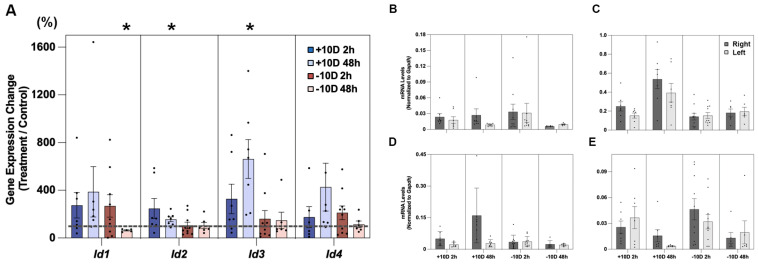
Defocus-induced changes in RPE *Id1-4* gene expression shown in (**A**), expressed as ratios of results from treated versus fellow eyes (%), for 2 or 48 h of +10 or −10 D lens wear (+10 D, 2 h, n = 7; +10 D, 48 h, n = 7; −10 D, 2 h, n = 10; and −10 D, 48 h, n = 6). RPE mRNA levels, normalizing to *Gapdh*, for both treated and control eyes are also shown for *Id1*, *Id2*, *Id3*, and *Id4* ((**B**–**E**), respectively). Note differences in Y-axis scales for individual panels, used to accommodate gene-dependent differences in expression levels. * *p* < 0.05.

Significant defocus treatment effects were recorded for three *Id* genes, as evidenced by differences in gene expression between treated and fellow eyes (Figure 4A and Table 6). With the +10 D lens treatment, the expression of both *Id2* and *Id3* genes was significantly upregulated after 48 h of treatment, and for *Id2*, by 156.8 ± 19.8% (*p* = 0.04, n = 7), and for *Id3*, by 661.8 ± 162.8% (*p* = 0.03, n = 7). For the −10 D lens, only the expression of *Id1* was significantly altered, being downregulated to 63.5 ± 5.0% after 48 h (*p* = 0.01, n = 6). mRNA levels, normalized to *Gapdh*, for the same four genes and treated and fellow eyes, are shown in Figure 4B–E and Table 7. Treatment-induced differential *Id* gene expression data, expressed as fold changes (log2), are provided in Supplemental Table S2 and Figure S2.

### 3.3. Id Gene Expression in the Choroid and Effects of Lens Treatment

**Figure 5:**
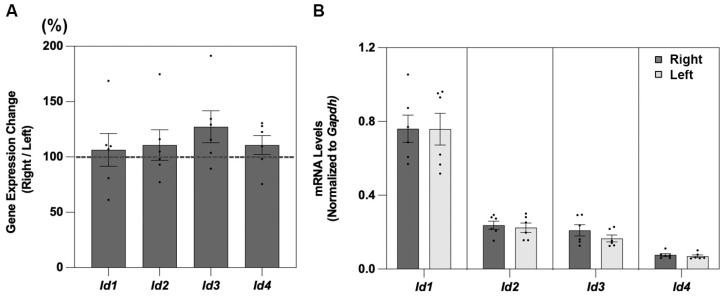
Choroidal *Id1-4* gene expression levels are shown in (**A**), as ratios of values for right versus left eyes of untreated chicks (%, n = 6), and mRNA levels, normalized to *Gapdh*, for the same eyes in (**B**).

For all four of the *Id* genes examined, expression reached detectable levels in choroidal tissue collected from untreated chicks (Figure 5 and Table 8). Here also, there were no significant differences in mRNA levels between their right and left eyes for any of the 4 *Ids*, of which *Id1* showed relatively higher expression than *Id2*, *Id3*, and *Id4* (Figure 5B).

**Figure 6:**
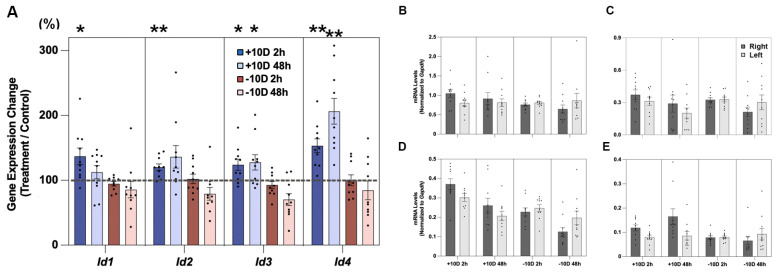
Choroidal *Id1-4* gene expression changes after 2 or 48 h of +10 or −10 D lens wear shown in (**A**), expressed as ratios of values for treated eyes versus control eyes (%, n = 10 for all treatment groups). Choroidal mRNA levels, normalized to *Gapdh*, are also shown for treated and control eyes, and *Id1*, *Id2*, *Id3*, and *Id4* ((**B**–**E**), respectively). Note differences in Y-axis scales in individual panels, used to accommodate gene-dependent differences in expression levels. * *p* < 0.05, ** *p* < 0.01.

Significant defocus-induced effects were recorded for all four genes, although mostly confined to the myopic defocus (+10 D lens) treatment, as apparent in Figure 6A and data summarized in Table 9. For the +10 D lens, all four *Id* genes were significantly upregulated after 2 h of treatment, i.e., for *Id1* by 137.0 ± 12.7% (*p* = 0.03, n = 10), *Id2* by 120.0 ± 5.1% (*p* = 0.008), *Id3* by 124.0 ± 8.7% (*p* = 0.02), and *Id4* by 153.2 ± 10.7% (*p* = 0.001). Both *Id3* and *Id4* genes also remained upregulated after the longer 48 h treatment, by 127.5 ± 11.8% (*p* = 0.04, n = 10) and 206.3 ± 19.9% (*p* = 0.001), respectively. In contrast, with the hyperopic (−10 D lens) treatment, none of the four genes showed significant, treatment-induced changes in expression. mRNA levels normalized to *Gapdh* for the same four genes, and treated and fellow eyes are shown in Figure 6B–E and summarized in Table 10. Differential *Id* gene expression data, expressed as fold changes, are also provided in Supplemental Table S3 and Figure S3.

## 4. Discussion

Our previous studies pointed to important roles for RPE-derived BMPs as critical signaling molecules in eye growth regulation in the chick model [18,19,20,21,22,23,24], with more supporting evidence implicating BMP2 emerging from our recent study involving contact lens-induced myopia in the guinea pig model, in which both *Bmp2* and *Id3* gene expression were found to be downregulated in RPE [19]. Specifically, the expressions of genes *Bmp2*, *4*, and *7* were all found to show optical defocus sign-dependent, bidirectional regulation, i.e., up- and downregulation in response to positive and negative defocusing lenses, respectively, even after as little as 2 h of exposure. Furthermore, BMPs were detected in all posterior ocular tissues, i.e., retina, RPE, choroid, and sclera [23,24], with the RPE being one of the major sources of synthesized BMPs. Further supporting evidence for roles of BMPs in eye growth regulation is provided by the results of the study reported here, which aimed to elucidate the downstream signaling pathways of BMPs, specifically by investigating defocus-induced differential expression of *Id* genes in retina, RPE, and choroid.

The functional roles of BMPs are known to be both tissue- and context-dependent. For example, BMPs have been shown to induce differentiation of stem cells, and have also been observed to inhibit proliferation of cancer cells [37,38]. Consistent with Ids being components of the BMP signaling pathways [28,31,32,33,34,39], related tissue- and context-dependent differences in function have also been reported. For example, in one study involving Id3, overexpression led to inhibition of cell proliferation [39], while in another study *Id* genes induced by BMPs were reported to suppress differentiation and increase stem cell renewal [40]. A number of other studies have specifically focused on the regulation and functions of BMP-Id signaling pathways in various ocular tissues [19,41,42,43,44]. In one such study, BMPs and BMP receptors were found to be co-localized with Id1 and Id3 in retinal progenitor cells in the adult mouse retina, with phosphorylation of Smad1/5/8 by BMP4 proposed as the trigger for upregulation of both Id1 and Id3 [44]. In yet another retinal study, BMP regulation of Id1 and Id3 expression was linked to glial differentiation [43]. In a third example, involving the more anteriorly located crystalline lens of the eye, Id2 and Id3, linked to TGF-β/BMP signaling, were assigned important coordinating roles in the control of cell differentiation and proliferation [41,42].

Given the diverse functional roles of and complicated interactions between BMPs and *Id* genes, and the wide distribution of the former in posterior ocular tissues, we expected and observed complex patterns of *Id* gene expression changes in the chick retina, RPE, and choroid in response to the applied optical defocus treatments. Overall, observed defocus-induced *Id* gene expression changes were more consistent in the choroid and RPE compared to the retina [23,24]. As shown previously, imposed myopic defocus (i.e., positive lens treatments) for as little as 2 or 48 h induced *Bmp2* gene upregulation in chick RPE, and in the current study, all 4 *Id* genes were also found to be upregulated in the choroid after 2 h exposure to myopic defocus. These results represent indirect evidence of BMP2 synthesis in response to imposed myopic defocus, presumably in the RPE, with subsequent secretion across its basal wall, leading in turn to *Id* gene expression upregulation in the choroid. Note that the attribution of RPE-derived BMPs to the differential *Id* gene expression in the choroid is tentative, with the potential influences of other factors, including intraocular pressure-related mechanical stretch of one or more posterior ocular tissues and altered choroidal blood flow, among possibilities warranting consideration [45,46,47]. For example, in a previous study involving chondrocytes, gene expression of *Ids* was reported to decrease under high hydrostatic pressure [48]. Furthermore, the expression of the membrane-type matrix metalloproteinase 1 (MT1-MMP) has been shown to decrease in proportion to the decrease in ID-1 protein levels in breast cancer cells [49], with altered gene expression of MT1-MMP also observed in the scleras of tree shrew eyes with lens-induced myopia [50]. As yet another example, administration of brimonidine to myopic guinea pig eyes not only lowered intraocular pressure, but it also increased choroidal thickness and blood flow, and upregulated MMP-2 expression in the RPE-choroid complex of treated eyes [51,52]. Thus, the possibility that differential *Id* gene expression may be affected by other local factors, including changes in choroidal thickness and scleral remodeling, cannot be ruled out.

In the current study, optical defocus-driven bidirectional changes in *Id* gene expression were also observed in RPE, with both *Id2* and *Id3* gene expression being upregulated and *Id1* gene expression downregulated, with positive and negative lens treatments, respectively. These changes are consistent with autocrine signaling in RPE [24]. Of note, recent studies using the lens-induced guinea pig myopia model also reported *Id3* gene expression to be downregulated in RPE after both 1 and 7 days of negative lens treatment [19,53]. Despite the differences in the identity of the differentially regulated *Ids* in the RPE of these chick and guinea pig defocus myopia models, that they both implicate Ids argues for further investigations to better understand the role of the Id family in the RPE and eye growth regulation.

The gene expression changes observed in the RPE and choroid contrast with the more complicated patterns of differential *Id* gene expression observed in the retina. Specifically, gene expression upregulation in response to the positive lens treatment was limited to the *Id2* gene, while the negative lens treatment had opposite effects on the expression of the *Id1* gene, which showed downregulation, compared to the *Id3* and *Id4* genes, which showed upregulation. While we also observed large variability in retinal *Id1* gene expression with the 2 h +10 D lens treatment, this appeared to reflect a single outlier, with an abnormally high level of gene expression upregulation. Overall, the complexity of retinal *Id* gene expression patterns likely reflects the large number and diverse range of retinal cell types, which can also be expected to vary significantly in their responses to imposed defocus treatment. Such complexities preclude meaningful speculation on the contributions of various retinal cell populations to the observed changes in *Id* gene expression and the possible influence of RPE-derived BMPs, secreted via their apical surface.

## 5. Conclusions

We observed significant changes in the expressions of one or more *Id* genes in chick retina, RPE, and choroid in response to short-term, optical defocus stimuli of opposite signs. The observed patterns of *Id* gene expression in the choroid under the different defocus conditions align well with our previous findings for BMPs in RPE, consistent with roles for one or more BMP-Id signaling pathways in the regulation of eye growth. Nonetheless, the complicated nature of the observed defocus-induced gene expression patterns points to additional, yet to be identified, influences on eye growth more generally. Further investigations into retina–RPE–choroid signaling pathways, both downstream and upstream, are needed to complete this picture, by ruling out the possibility of as yet unidentified key molecules, with the hope that one or more of the final list of key molecules could serve as targets for novel ophthalmic anti-myopia therapeutic interventions.

## Figures and Tables

**Table 1:** Primer information for chick *Id1*, *Id2*, *Id3*, and *Id4*.

Gene	NCBI Access Number	Sequences (5′–3′)	Efficiency	Amplicon
*Id1*	NM_204590.2	Forward: 5′-CGCGGCTAGTAACCTTCTCAGA-3′ Reverse: 5′-TTCTCCGGCATCATTGTAATATACA-3′	91.8%	69 bp
*Id2*	NM_205002.1	Forward: 5′-CCCTACAGGCAGCCGAGTT-3′ Reverse: 5′-TCAGCCACAGAGCGCTTTG-3	97.7%	65 bp
*Id3*	NM_204589.1	Forward:5′-TGCTCCAAAGACGAGAGAAGTTT-3′ Reverse: 5′-TGTTTGCTAATCGGCACTGATG-3′	94.4%	64 bp
*Id4*	NM_204282.1	Forward: 5′-TCCCTGCAGGAATGTTGCA-3′ Reverse: 5′ ATCTCTCTATGTACACGGTATGAAATGTC-3′	97.2%	68 bp
*Gapdh*	NM_204305.1	Forward: 5′-AGATGCAGGTGCTGAGTATGTTG-3′ Reverse: 5′-GATGAGCCCCAGCCTTCTC-3′	95.6%	71 bp

**Table 2:** Retinal *Id1-4* gene expression for right and left eyes of untreated chicks (SEM in brackets).

	Right (n = 5)	Left (n = 5)	% Change (Right/Left)
*Id1*	1.2 × 10^−4^ [6.0 × 10^−5^]	1.2 × 10^−4^ [5.5 × 10^−5^]	109.2 [10.3]
*Id2*	1.0 × 10^−2^ [9.6 × 10^−4^]	1.0 × 10^−2^ [1.2 × 10^−3^]	110.7 [9.5]
*Id3*	8.8 × 10^−4^ [5.4 × 10^−5^]	8.2 × 10^−4^ [8.2 × 10^−5^]	110.4 [7.5]
*Id4*	6.7 × 10^−2^ [7.4 × 10^−3^]	6.3 × 10^−2^ [4.3 × 10^−3^]	105.5 [5.1]

**Table 3:** Retinal *Id1-4* gene expression changes induced by 2 or 48 h of +10 and −10 D lens wear, reported as mean ratios (%) of expression levels in treated versus control eyes (SEM in brackets).

	*Id1*	*Id2*	*Id3*	*Id4*
+10 D 2 h (n = 11)	331.3 [216.8]	123.3 [7.8] *	129.1 [17.1]	112.1 [5.7]
+10 D 48 h (n = 6)	68.4 [13.0]	131.2 [9.2] *	117.4 [9.9]	107.2 [5.3]
−10 D 2 h (n = 11)	80.9 [10.4]	107.4 [5.9]	116.2 [6.7] *	108.5 [6.0]
−10 D 48 h (n = 8)	75.6 [4.9] **	117.7 [19.5]	104.0 [13.2]	116.3 [6.7] *

* *p* < 0.05, ** *p* < 0.01.

**Table 4:** Retinal *Id1-4* gene expression levels in treated and control eyes, after 2 or 48 h of +10 or −10 D lens wear, normalized to *Gapdh* (SEM in brackets).

	*Id1*		*Id2*		*Id3*		*Id4*	
	Treatment	Control	Treatment	Control	Treatment	Control	Treatment	Control
+10 D 2 h (n = 11)	6.6 × 10^−4^ [4.5 × 10^−4^]	1.7 × 10^−4^ [3.4 × 10^−5^]	1.1 × 10^−2^ [1.0 × 10^−3^]	9.2 × 10^−3^ [8.2 × 10^−4^]	1.2 × 10^−3^ [1.6 × 10^−4^]	1.0 × 10^−3^ [1.2 × 10^−4^]	8.1 × 10^−2^ [7.3 × 10^−3^]	7.2 × 10^−2^ [5.8 × 10^−3^]
+10 D 48 h (n = 6)	2.2 × 10^−4^ [1.1 × 10^−5^]	4.4 × 10^−4^ [1.3 × 10^−4^]	1.1 × 10^−2^ [1.8 × 10^−3^]	7.8 × 10^−3^ [1.1 × 10^−3^]	9.5 × 10^−4^ [8.4 × 10^−5^]	8.3 × 10^−4^ [9.4 × 10^−5^]	8.6 × 10^−2^ [2.6 × 10^−3^]	8.1 × 10^−2^ [3.5 × 10^−3^]
−10 D 2 h (n = 11)	1.5 × 10^−4^ [4.0 × 10^−5^]	2.0 × 10^−4^ [4.6 × 10^−5^]	9.3 × 10^−3^ [8.0 × 10^−4^]	8.8 × 10^−3^ [7.2 × 10^−4^]	1.0 × 10^−3^ [7.5 × 10^−5^]	8.7 × 10^−4^ [1.0 × 10^−4^]	8.2 × 10^−2^ [9.0 × 10^−3^]	7.5 × 10^−2^ [6.6 × 10^−3^]
−10 D 48 h (n = 8)	2.4 × 10^−4^ [2.2 × 10^−5^]	3.1 × 10^−4^ [2.8 × 10^−5^]	1.1 × 10^−2^ [1.6 × 10^−3^]	1.3 × 10^−2^ [4.0 × 10^−3^]	7.2 × 10^−4^ [1.0 × 10^−4^]	7.3 × 10^−4^ [1.0 × 10^−4^]	1.0 × 10^−1^ [3.1 × 10^−3^]	8.7 × 10^−2^ [3.1 × 10^−3^]

**Table 5:** RPE *Id1-4* gene expression levels for right and left eyes of untreated chicks (SEM in brackets).

	Right (n = 3)	Left (n = 3)	% Change (Right/Left)
*Id1*	5.3 × 10^−2^ [3.2 × 10^−2^]	1.4 × 10^−2^ [3.8 × 10^−3^]	399.0 [181.8]
*Id2*	1.6 × 10^−1^ [2.1 × 10^−2^]	1.5 × 10^−1^ [3.7 × 10^−2^]	128.3 [52.8]
*Id3*	2.3 × 10^−2^ [7.0 × 10^−3^]	1.7 × 10^−2^ [3.4 × 10^−3^]	150.4 [49.2]
*Id4*	6.7 × 10^−2^ [2.6 × 10^−2^]	6.3 × 10^−2^ [1.7 × 10^−2^]	118.8 [41.2]

**Table 6:** Changes in RPE *Id1-4* gene expression levels induced by 2 or 48 h of +10 and −10 D lens wear, reported as mean ratios (%) of expression in treated versus control eyes (SEM in brackets).

	*Id1*	*Id2*	*Id3*	*Id4*
+10 D 2 h (n = 7)	274.4 [106.0]	247.7 [84.2]	327.4 [124.1]	190.5 [76.3]
+10 D 48 h (n = 7)	387.7 [211.1]	156.8 [19.8] *	661.8 [162.8] *	426.7 [200.8]
−10 D 2 h (n = 10)	268.7 [94.2]	105.6 [27.1]	161.2 [69.0]	211.2 [56.8]
−10 D 48 h (n = 6)	63.5 [5.0] *	104.9 [26.5]	147.5 [68.8]	114.4 [27.7]

* *p* < 0.05.

**Table 7:** RPE *Id1-4* gene expression levels for treated and control eyes, after +10 or −10 D lens wear for 2 or 48 h, normalized to *Gapdh* (SEM in brackets).

	*Id1*		*Id2*		*Id3*		*Id4*	
	Treatment	Control	Treatment	Control	Treatment	Control	Treatment	Control
+10 D 2 h (n = 7)	2.4 × 10^−2^ [6.4 × 10^−3^]	1.8 × 10^−2^ [6.3 × 10^−3^]	2.5 × 10^−1^ [4.9 × 10^−2^]	1.5 × 10^−1^ [2.7 × 10^−2^]	5.0 × 10^−2^ [1.3 × 10^−2^]	2.2 × 10^−2^ [4.2 × 10^−3^]	2.6 × 10^−2^ [7.0 × 10^−3^]	3.7 × 10^−2^ [1.3 × 10^−2^]
+10 D 48 h (n = 7)	2.7 × 10^−2^ [1.2 × 10^−2^]	8.7 × 10^−3^ [1.0 × 10^−3^]	5.4 × 10^−1^ [1.0 × 10^−1^]	3.9 × 10^−1^ [9.8 × 10^−2^]	1.6 × 10^−1^ [4.9 × 10^−2^]	2.8 × 10^−2^ [6.6 × 10^−3^]	1.6 × 10^−2^ [7.1 × 10^−3^]	3.7 × 10^−3^ [3.1 × 10^−4^]
−10 D 2 h (n = 10)	3.3 × 10^−2^ [1.5 × 10^−2^]	3.1 × 10^−2^ [1.8 × 10^−2^]	1.4 × 10^−1^ [3.6 × 10^−2^]	1.5 × 10^−1^ [3.0 × 10^−2^]	3.4 × 10^−2^ [1.0 × 10^−2^]	3.7 × 10^−2^ [7.6 × 10^−3^]	4.6 × 10^−2^ [1.2 × 10^−2^]	3.2 × 10^−2^ [8.4 × 10^−3^]
−10 D 48 h (n = 6)	5.9 × 10^−3^ [4.7 × 10^−4^]	9.5 × 10^−3^ [9.9 × 10^−4^]	1.8 × 10^−1^ [4.0 × 10^−2^]	1.9 × 10^−1^ [4.4 × 10^−2^]	2.4 × 10^−2^ [7.0 × 10^−3^]	2.0 × 10^−2^ [2.3 × 10^−3^]	1.3 × 10^−2^ [6.4 × 10^−3^]	2.0 × 10^−2^ [1.3 × 10^−2^]

**Table 8:** Choroidal *Id1-4* gene expression in the right and left eyes of untreated chicks (SEM in brackets).

	Right (n = 6)	Left (n = 6)	% Change (Right/Left)
*Id1*	7.6 × 10^−1^ [7.4 × 10^−2^]	7.6 × 10^−1^ [8.6 × 10^−2^]	106.4 [14.8]
*Id2*	2.4 × 10^−1^ [2.2 × 10^−2^]	2.2 × 10^−1^ [2.6 × 10^−2^]	110.7 [13.8]
*Id3*	2.1 × 10^−1^ [3.0 × 10^−2^]	1.7 × 10^−1^ [1.9 × 10^−2^]	127.3 [14.5]
*Id4*	7.6 × 10^−2^ [7.7 × 10^−3^]	7.0 × 10^−2^ [7.1 × 10^−3^]	110.8 [8.6]

**Table 9:** Choroidal *Id1-4* gene expression changes induced by +10 and −10 D lens treatments, reported as mean ratios (%) of expression in treated versus control eyes (SEM in brackets).

	*Id1*	*Id2*	*Id3*	*Id4*
+10 D 2 h (n = 10)	137.0 [12.7] *	120.0 [5.1] **	124.0 [8.7] *	153.2 [10.7] **
+10 D 48 h (n = 10)	112.4 [10.2]	136.1 [17.4]	127.5 [11.8] *	206.3 [19.9] **
−10 D 2 h (n = 10)	94.6 [3.4]	102.0 [7.3]	92.6 [5.4]	100.0 [8.4]
−10 D 48 h (n = 10)	85.5 [12.6]	78.9 [9.9]	70.2 [9.2]	84.4 [14.3]

* *p* < 0.05, ** *p* < 0.01.

**Table 10:** Choroidal *Id1-4* gene expression in treated and control eyes, after 2 or 48 h of +10 or −10 D lens wear, normalized to *Gapdh* (SEM in brackets).

	*Id1*		*Id2*		*Id3*		*Id4*	
	Treatment	Control	Treatment	Control	Treatment	Control	Treatment	Control
+10 D 2 h (n = 10)	1.0 [1.0 × 10^−1^]	8.0 × 10^−1^ [8.3 × 10^−2^]	3.7 × 10^−1^ [4.6 × 10^−2^]	3.1 × 10^−1^ [3.8 × 10^−2^]	3.7 × 10^−1^ [2.8 × 10^−2^]	3.0 × 10^−1^ [2.0 × 10^−2^]	1.2 × 10^−1^ [1.3 × 10^−2^]	8.0 × 10^−2^ [8.9 × 10^−3^]
+10 D 48 h (n = 10)	9.1 × 10^−1^ [1.5 × 10^−1^]	8.1 × 10^−1^ [9.5 × 10^−2^]	2.9 × 10^−1^ [8.0 × 10^−2^]	2.0 × 10^−1^ [4.6 × 10^−2^]	2.6 × 10^−1^ [3.7 × 10^−2^]	2.1 × 10^−1^ [2.4 × 10^−2^]	1.7 × 10^−1^ [3.2 × 10^−2^]	8.5 × 10^−2^ [1.7 × 10^−2^]
−10 D 2 h (n = 10)	7.6 × 10^−1^ [4.1 × 10^−2^]	8.1 × 10^−1^ [4.1 × 10^−2^]	3.2 × 10^−1^ [1.9 × 10^−2^]	3.3 × 10^−1^ [2.2 × 10^−2^]	2.3 × 10^−1^ [2.1 × 10^−2^]	2.5 × 10^−1^ [1.8 × 10^−2^]	7.9 × 10^−2^ [7.4 × 10^−3^]	8.0 × 10^−2^ [5.3 × 10^−3^]
−10 D 48 h (n = 10)	6.5 × 10^−1^ [1.0 × 10^−1^]	8.6 × 10^−1^ [1.9 × 10^−1^]	2.1 × 10^−1^ [4.6 × 10^−2^]	3.0 × 10^−1^ [6.8 × 10^−2^]	1.3 × 10^−1^ [2.2 × 10^−2^]	2.0 × 10^−1^ [3.5 × 10^−2^]	6.5 × 10^−2^ [1.7 × 10^−2^]	9.2 × 10^−2^ [2.4 × 10^−2^]

## Data Availability

The original contributions presented in this study are included in the article/Supplementary Material. Further inquiries can be directed to the corresponding author.

## Supplementary Materials

The following supporting information can be downloaded at: https://www.mdpi.com/article/10.3390/cells14231883/s1, Table S1. Retinal *Id1-4* gene expression changes induced by 2 or 48 h of +10 and −10 D lens treatments, reported as fold changes (Log2) in treated relative to control eyes (treated/control); SEM in brackets. Figure S1: Retinal *Id1-4* gene expression changes, expressed as fold (Log2) of values for treated relative to control eyes after 2 or 48 h of +10 or −10 D lens treatment (+10 D 2 h, n = 11; +10 D 48 h, n = 6; −10 D 2 h, n = 11; −10 D 48 h, n = 8). * *p* < 0.05, ** *p* < 0.01. Table S2. Changes in RPE *Id1-4* gene expression levels induced by 2 or 48 h of +10 and −10 D lens treatments, reported as fold changes (Log2) in treated relative to control eyes (treated/control); SEM in brackets. Figure S2: RPE *Id1-4* gene expression changes, expressed as fold (Log2) of values for treated relative to control eyes after 2 or 48 h of +10 or −10 D lens treatment (+10 D 2 h, n = 7; +10 D 48 h, n = 7; −10 D 2 h, n = 10; −10 D 48 h, n = 6). * *p* < 0.05. Table S3. Choroidal *Id*1-4 gene expression changes induced by +10 and −10 D lens treatments, reported as fold changes (Log2) in treated relative to control eyes (treated/control); SEM in brackets. Figure S3: Choroidal *Id1-4* gene expression changes, expressed as fold (Log2) of values for treated relative to control eyes after 2 or 48 h of +10 or −10 D lens treatment (n = 10 for all treatment groups). * *p* < 0.05, ** *p* < 0.01. Figure S4: *Id1-4* gene expression levels in retina, RPE, and choroid from untreated chicks.

## Abbreviations

The following abbreviations are used in this manuscript:

BMP	Bone Morphogenetic Protein
Gapdh	Glyceraldehyde-3-phosphate dehydrogenase
*Id*	Inhibitors of DNA-binding gene
RPE	Retinal Pigment Epithelium
TGF-β	Transforming Growth Factor Beta
