# Supplementary material for: Effects of Imposed Defocus on Inhibitor of DNA-Binding Gene Expression in Chick Posterior Ocular Tissues
Source: Cells. 2025 Nov 27;14(23):1883. doi: 10.3390/cells14231883 (PMC12691209; doi:10.3390/cells14231883)
Supplement: Supplementary file 1 [file cells-14-01883-s001.zip › cells-3866952-supplementary.pdf]

Supplemental Materials

Table S1. Retinal *Id1-4* gene expression changes induced by 2 or 48 h of +10 and -10 D lens treatments, reported as fold changes (Log2) in treated relative to control eyes (treated/control); SEM in brackets

	Id1	*Id2*	*Id3*	*Id4*
+10 D 2 h (n = 11)	0.11 [0.56]	0.27 [0.09]*	0.27 [0.16]	0.15 [0.07]
+10 D 48 h (n = 6)	-0.73 [0.36]	0.37 [0.11]*	0.21 [0.11]	0.09 [0.07]
-10 D 2 h (n = 11)	-0.41 [0.17]	0.08 [0.09]	0.19 [0.08]*	0.09 [0.09]
-10 D 48 h (n = 8)	-0.42 [0.09]**	0.05 [0.31]	-0.03 [0.20]	0.20 [0.08]*

* $p < 0.05$, ** $p < 0.01$

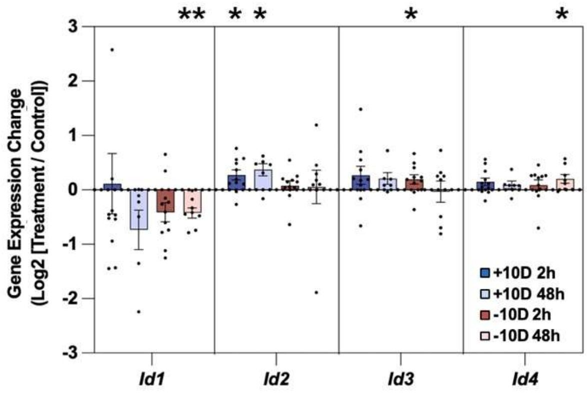

Figure S1: Retinal *Id1-4* gene expression changes, expressed as fold (Log2) of values for treated relative to control eyes after 2 or 48 h of +10 or -10 D lens treatment (+10 D 2 h, n = 11; +10 D 48 h, n = 6; -10 D 2 h, n = 11; -10 D 48 h, n = 8). * $p < 0.05$, ** $p < 0.01$.

Table S2. Changes in RPE *Id1-4* gene expression levels induced by 2 or 48 h of +10 and -10 D lens treatments, reported as fold changes (Log2) in treated relative to control eyes (treated/control); SEM in brackets

	Id1	*Id2*	*Id3*	*Id4*
+10 D 2 h (n = 10)	0.82 [0.57]	0.80 [0.51]	1.03 [0.59]	0.002 [0.76]
+10 D 48 h (n = 10)	1.18 [0.53]	0.58 [0.19] *	2.42 [0.41] *	1.39 [0.54]
-10 D 2 h (n = 10)	0.14 [0.88]	-0.32 [0.36]	-0.31 [0.56]	0.45 [0.50]
-10 D 48 h (n = 10)	-0.67 [0.12] *	-0.14 [0.34]	0.05 [0.48]	0.006 [0.32]

* $p < 0.05$, ** $p < 0.01$

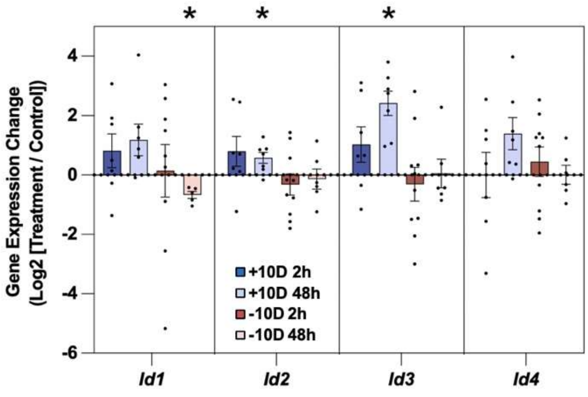

Figure S2: RPE *Id1-4* gene expression changes, expressed as fold (Log2) of values for treated relative to control eyes after 2 or 48 h of +10 or -10 D lens treatment (+10 D 2 h, n = 7; +10 D 48 h, n = 7; -10 D 2 h, n = 10; -10 D 48 h, n = 6). * $p < 0.05$.

Table S3. Choroidal *Id*1-4 gene expression changes induced by +10 and -10 D lens treatments, reported as fold changes (Log2) in treated relative to control eyes (treated/control); SEM in brackets

	Id1	*Id2*	*Id3*	*Id4*
+10 D 2 h (n = 7)	0.40 [0.13] *	0.25 [0.06] **	0.28 [0.10] *	0.58 [0.10] **
+10 D 48 h (n = 7)	0.10 [0.15]	0.36 [0.16]	0.30 [0.12] *	0.98 [0.14] **
-10 D 2 h (n = 10)	-0.09 [0.05]	-0.004 [0.11]	-0.13 [0.09]	-0.04 [0.12]
-10 D 48 h (n = 6)	-0.36 [0.22]	-0.43 [0.17]	-0.64 [0.22]	-0.43 [0.25]

* $p < 0.05$, ** $p < 0.01$

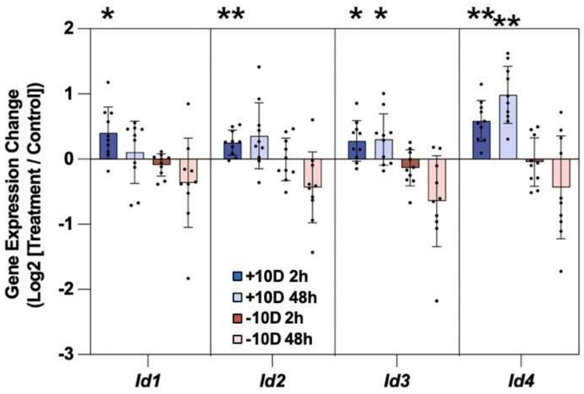

Figure S3: Choroidal *Id1-4* gene expression changes, expressed as fold (Log2) of values for treated relative to control eyes after 2 or 48 h of +10 or -10 D lens treatment (n = 10 for all treatment groups). * $p < 0.05$, ** $p < 0.01$.

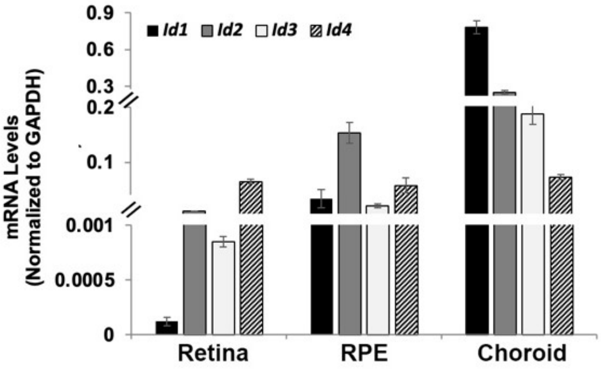

Figure S4: *Id1-4* gene expression levels in retina, RPE, and choroid from untreated chicks.
